# Alterations in immune cell phenotype and cytotoxic capacity in HER2+ breast cancer patients receiving HER2-targeted neo-adjuvant therapy

**DOI:** 10.1038/s41416-023-02375-y

**Published:** 2023-07-28

**Authors:** Nicola Gaynor, Alfonso Blanco, Stephen F. Madden, Barry Moran, Jean M. Fletcher, Damien Kaukonen, Javier Sánchez Ramírez, Alex J. Eustace, Martina S. J. McDermott, Alexandra Canonici, Sinead Toomey, Ausra Teiserskiene, Bryan T. Hennessy, Norma O’Donovan, John Crown, Denis M. Collins

**Affiliations:** 1 Cancer Biotherapeutics Research Group, National Institute for Cellular Biotechnology, Dublin City University, Dublin, Ireland; 2 Flow Cytometry Core Technology, UCD Conway Institute, University College Dublin, Dublin, Ireland; 3 Data Science Centre, School of Population Heath Sciences, RCSI University of Medicine and Health Sciences, Dublin, Ireland; 4 School of Biochemistry and Immunology, Trinity Biomedical Sciences Institute, Trinity College Dublin, Dublin, Ireland; 5 School of Medicine, Trinity Biomedical Sciences Institute, Trinity College Dublin, Dublin, Ireland; 6 School of Biotechnology, Dublin City University, Dublin, Ireland; 7 National Institute for Cellular Biotechnology, Dublin City University, Dublin, Ireland; 8 Medical Oncology Group, Department of Molecular Medicine, Royal College of Surgeons in Ireland, Dublin, Ireland; 9 Cancer Trials Ireland, RCSI House, 121 St. Stephen’s Green, Dublin, Ireland; 10 Department of Medical Oncology, St Vincent’s University Hospital, Dublin, Ireland

**Keywords:** Breast cancer, Translational immunology, Cancer therapy

## Abstract

**Background:**

The phase II neo-adjuvant clinical trial ICORG10-05 (NCT01485926) compared chemotherapy in combination with trastuzumab, lapatinib or both in patients with HER2+ breast cancer. We studied circulating immune cells looking for alterations in phenotype, genotype and cytotoxic capacity (direct and antibody-dependent cell-mediated cytotoxicity (ADCC)) in the context of treatment response.

**Methods:**

Peripheral blood mononuclear cells (PBMCs) were isolated from pre- (*n* = 41) and post- (*n* = 25) neo-adjuvant treatment blood samples. Direct/trastuzumab-ADCC cytotoxicity of patient-derived PBMCs against K562/SKBR3 cell lines was determined ex vivo. Pembrolizumab was interrogated in 21 pre-treatment PBMC ADCC assays. Thirty-nine pre-treatment and 21 post-treatment PBMC samples were immunophenotyped. Fc receptor genotype, tumour infiltrating lymphocyte (TIL) levels and oestrogen receptor (ER) status were quantified.

**Results:**

Treatment attenuated the cytotoxicity/ADCC of PBMCs. CD3+/CD4+/CD8+ T cells increased following therapy, while CD56+ NK cells/CD14+ monocytes/CD19+ B cells decreased with significant post-treatment immune cell changes confined to patients with residual disease. Pembrolizumab-augmented ex vivo PBMC ADCC activity was associated with residual disease, but not pathological complete response. Pembrolizumab-responsive PBMCs were associated with lower baseline TIL levels and ER+ tumours.

**Conclusions:**

PBMCs display altered phenotype and function following completion of neo-adjuvant treatment. Anti-PD-1-responsive PBMCs in ex vivo ADCC assays may be a biomarker of treatment response.

## Introduction

Human epidermal growth factor receptor 2-positive (HER2+) breast cancer accounts for approximately 20% of all breast cancers [1]. HER2+ breast cancer is characterised by amplification of the *ERBB2* gene (chromosome assignment 17q12), leading to overexpression of the HER2 protein [1]. This results in increased activation of cell growth and proliferation pathways including the PI3K and MAPK pathways [2]. HER2+ breast tumours are highly proliferative and aggressive, and had a poor prognosis before the advent of HER2-targeted therapies [3].

Trastuzumab was the first HER2-targeted therapy approved for the treatment of HER2+ breast cancer. Trastuzumab targets a juxtamembrane epitope in subdomain IV of the extracellular portion of the HER2 protein. Once bound, trastuzumab suppresses HER2 intracellular signalling, and trastuzumab’s human IgG1 isotype framework was deliberately designed to elicit antibody-dependent cell-mediated cytotoxicity (ADCC) [4]. ADCC involves Fc receptor (e.g. FCGR3A/CD16) expressing effector cells recognising autologous or therapeutic antibodies bound to a target cell and inducing cell death by triggering apoptosis through the perforin/granzyme system [5]. Examples of ADCC capable immune cells that engage IgG antibodies like trastuzumab include NK cells and monocytes [6]. Clynes et al. demonstrated severely reduced efficacy of trastuzumab in CD16-knockout mice and the mutation of the Fc region of trastuzumab reducing its interaction with activating Fc receptors (FCGRs) [7]. In addition, FCGR single nucleotide polymorphisms (SNPs) have been associated with altered (increased or attenuated) immune cell ADCC capacity, and pathological complete response (pCR) to trastuzumab-based therapy [8]. It has also been reported that in vitro, trastuzumab-mediated ADCC correlates with response to trastuzumab treatment using patient immune cells [9–11]. Recent clinical studies have shown that variable ADCC capacity of HER2-targeted therapeutic antibodies is associated with patient outcome [12, 13].

Lapatinib is a small molecule tyrosine kinase inhibitor (TKI) that targets EGFR and HER2 and it is approved for use in combination with capecitabine as second-line therapy for metastatic HER2+ breast cancer that is refractory to trastuzumab, or to treat advanced hormone receptor positive HER2+ breast cancer in combination with letrozole [14, 15]. Combining antibody therapies and TKIs results in synergy through more complete inhibition of HER2 signalling, the additional immune engagement mediated by the antibody therapies, and the potential for TKIs to overcome resistance mechanisms such as constitutively activated p95 HER2 and HER-family compensation mechanisms [2, 4, 16]. The ICORG 10-05 clinical trial was initiated to investigate the efficacy of docetaxel (T)/carboplatin (C) with trastuzumab (H) or lapatinib (L) or the combination (HL) based on pCR to neo-adjuvant therapy in HER2+ breast cancer patients. In the intent-to-treat population, no significant differences between pCR were detected for TCH (48%) vs TCHL (44%) [17].

Other clinical trials investigating the combination of trastuzumab and lapatinib in the neo-adjuvant setting have been conducted: CHER-LOB [18], Neo-ALTTO [19], CALGB40601 [20], NSABP-B41 [21], TRIO-B07 [22], EORTC100451 [23] and LPT109096 [24]. Only two of these seven trials (Neo-ALTTO, CHER-LOB) showed a statistically significant improvement in pCR for the combination. Discordant results could be explained through multiple differences between the trials including treatment scheduling, the chemotherapy backbone used and definition of pCR. Extensive translational studies were carried out in these trials to identify potential biomarkers of pCR that could be used to enrich patient cohorts for treatment de-escalation studies, or by contrast, new treatments for innately resistant patient subgroups. Tumour molecular profiling has revealed lower ER expression, higher levels of HER2-amplification and the HER2-enriched PAM50 molecular subtype as indicators of higher rates of pCR [20, 25, 26]. Multiple immune-related biomarkers including tumour infiltrating lymphocyte (TIL) levels and immune gene signatures that identify patients with increased rates of pCR have also been reported [25, 27–32]. Despite these significant associations with pCR outcomes in HER2+ early disease, no validated biomarker of pCR with sufficient accuracy to function on an individual patient basis has yet been identified. We hypothesised that immune response might be a determinant of pCR to HER2-targeted treatment strategies. This study utilises translational clinical material from the ICORG 10-05 clinical trial to examine this hypothesis using data on circulating and tumour infiltrating immune cells. The direct cytotoxicity/ADCC capacity +/− pembrolizumab, immunophenotype composition (CD56+, CD14+, CD3+, CD4+, CD8+, CD19+, PD-1+), and FCGR genotype of circulating immune cells has not been examined previously in the context of pCR to neo-adjuvant chemotherapy/HER2-targeted therapy treatment.

## Materials and methods

### Cell lines and reagents

Cell lines were cultured at 37 °C/5% CO_2_ without antibiotics and with routine monitoring for *Mycoplasma* contamination. HER2+/MHC Class I+ breast cancer cell line SKBR3 and HER2-negative/MHC Class I-deficient/direct cytotoxicity-sensitive leukaemic cell line K562 were obtained from the American Type Culture Collection (ATCC) [33–35] Cell lines were maintained in RPMI 1640/10% heat-inactivated foetal bovine serum (HI FBS). Cell lines were authenticated by STR DNA profiling (Source Bio-science). Trastuzumab and pembrolizumab were obtained from St. Vincent’s University Hospital pharmacy, Dublin, Ireland.

### Patient population and sampling time points

HER2+ breast cancer patients (*n* = 88, all female) were enrolled in the multi-centre, Phase II ICORG 10-05 (NCT01485926) neo-adjuvant clinical trial [17]. Patients were randomised to one of three trial arms to receive T (75 mg/m^2^), C (AUC 6) and H (8 mg/kg loading dose and 6 mg/kg for subsequent six cycles) and/or L (1000 mg daily until 1 week prior to surgery). GCSF was mandatory for all patients as primary prophylaxis for febrile neutropenia while receiving chemotherapy (24 h after chemotherapy received), as was a prophylactic steroid regimen (dexamethasone) prior to each dose of docetaxel. Following surgery, patients completed a total of 1 year of trastuzumab from first dose of trastuzumab. The TCL arm was discontinued early due to preliminary results of the NCIC CTG MA.31 trial that showed lapatinib alone to be inferior to trastuzumab, or trastuzumab and lapatinib in relation to patient survival outcomes [36]. The primary endpoint was pCR. Secondary endpoints were to assess the clinical response rate and overall response rate by treatment arm, and to investigate potential markers of response to trastuzumab- and lapatinib-based chemotherapy. Blood samples were taken from patients before cycle 1 of treatment (pre-treatment) and after they completed cycle 6 of neo-adjuvant treatment (post-treatment), but before surgery. pCR was defined as no residual invasive tumour in the breast or lymph nodes at surgery. The No pCR cohort in this study consists of patients classified as partial responders and non-responders.

### Sample processing

Blood was collected in EDTA blood tubes (BD Vacutainer #367525) and processed within 4 h of blood draw. PBMCs were isolated using Ficoll-Paque (ThermoFisher 11778538)-based density centrifugation. PBMCs were frozen in vials at 1 × 10^7^ cells/mL in HI FBS (Sigma Aldrich F9665) containing 5% DMSO (Sigma Aldrich D2650) following three wash steps in RPMI 1640/10% HI FBS/0.5% pen/strep (Gibco 15140122). PBMCs were stored in liquid nitrogen. PBMCs were slowly revived and washed in pre-warmed RPMI 1640/10% HI FBS/0.5% pen/strep. Revived PBMCs were incubated at 37 °C for 4-5 h before being checked for viability using Guava Viacount (Luminex 4000-0041) on a Guava Easycyte flow cytometer (Luminex).

### Immune cytotoxicity assays

The immune cytotoxicity assays described are based on flow cytometry-based protocols utilised previously, involving carboxyfluorescein succinimidyl ester (CFSE) (Sigma Aldrich 21888) staining of target cells and the use of aminoactinomycin-D (7AAD) (Sigma Aldrich 9400) as the membrane permeable dead cell dye [34, 35, 37]. The plates were read on a Guava Easycyte flow cytometer using the Guava InCyte program (Luminex) to determine the viability of the target cell population. Due to an inverse correlation between cytotoxicity and viability, EC samples with a viability of less than 70% were excluded from immune cytotoxicity analysis in line with previous studies [38]. Post-treatment TCL arm samples were utilised as a control for post-treatment TCH/TCHL arm samples to ensure there was no residual trastuzumab contamination present. Control wells included basal cell death +/−trastuzumab and/or pembrolizumab, 100% TC dead cell controls, ECs only and a negative control for ADCC (CD20-specific rituximab, 10 µg/mL). The plates were read on a Guava Easycyte flow cytometer using the Guava InCyte program to determine the viability of the TC population. A detailed description of the assays and the calculations used to determine the presented values can be found in Supplementary material.

### Fc gamma receptor genotyping

Patient samples (*n* = 44) were genotyped for Fc gamma receptor (FCGR) single nucleotide polymorphisms (SNPs) to control for associated population variances in ADCC. Germline DNA was extracted from PBMCs for 41 patients and DNA was also obtained from formalin-fixed paraffin-embedded (FFPE) tumour samples for 3 further patients. The SNP status (homozygous for reference allele, heterozygous, homozygous for minor allele) for four SNPs, rs1801274, rs396991, rs428888, and rs10917661, was determined using Agena MassArray technology (Agena Bioscience, San Diego, CA, USA). FCGR2B rs10917661 lost a sample due to a read failure. Additional ICORG 10-05 patient whole exome sequencing SNP data on rs1050501 (*n* = 13) was available from an existing dataset [39].

### Peripheral blood immunophenotyping

Healthy volunteer and patient PBMCs were isolated, thawed and counted as previously described. PBMC samples were brought to a concentration of 3 × 10^6^ cells/100 µL. An optimised IM Duraclone tube (Beckman Coulter B53309) was used to phenotype cell types. Titration and compensation were carried out for PD-1 antibody (BioLegend B36123) using PBMCs from patients and healthy volunteers. Fluorescence minus one (FMO) controls were included for gating purposes. PBMCs were incubated with 2.5 µg/1 × 10^6^ cells of human Fc block (BD Biosciences 564219). 100 µL PBMCs were added to an IM Duraclone tube followed by PD-1 antibody, along with a zombie viability dye (BioLegend B423107). Red blood cell lysis reagent (Beckman Coulter A09777) was added to the tube. The tubes were then incubated in the dark for 15 min at room temperature. The cells were centrifuged and resuspended in PBS containing 0.8% fixative solution (Beckman Coulter 8546859). Samples were stored overnight at 4 °C and analysed using the CytoFLEX LX (Beckman Coulter). CytoFLEX Daily QC was run to check instrument performance as per manufacturer specifications. Analysis was carried out using FCS Express 6.06.0022 software. Gating utilised FMO controls and the recommended Duraclone gating strategy (Beckman Coulter). Please see the Minimum information on a flow cytometry experiment document in Supplementary Material. Samples were run over 2 separate days. Principal component analysis was used to ensure consistency in results between runs. Technical issues relating to detection channels resulted in Day 2 CD56 and PD-1 staining being unavailable.

### Tumour infiltrating lymphocytes

Pre-treatment and on-treatment (20 days post cycle 1) stromal, tumour and overall (stromal + tumour) infiltrating lymphocyte counts for ICORG 10-05 were determined previously [40]. The current study presents pre-treatment FFPE baseline biopsy TIL counts from a subset of the published dataset corresponding to the patient PBMC samples examined for response to pembrolizumab in vitro. Counts were assessed by immunohistochemistry of CD45+ (Dako, Clones 2B11+PD7/26, M0701) and cytokeratin AE1/3 (Dako, Clone AE1/3, M3515) on FFPE tumour sections. A lymphocyte was defined as a TIL if it was in direct contact with an invasive tumour epithelial cell and stromal lymphocytes were defined based on dispersion within the stroma and no contact with the tumour epithelium [40].

### Ethical approval

The ICORG 10-05 clinical trial was conducted in accordance with the Declaration of Helsinki. The trial was approved by the relevant ethics committees/institutional review boards and health authorities at all participating sites in Ireland under the direction of the study sponsor, the All-Ireland Clinical Oncology Research Group (ICORG). Informed, written consent was obtained from all participants. Healthy volunteer samples were collected under DCU Ethics Committee approval with informed, written consent obtained from all volunteers.

### Statistical analysis

A pooled Student’s *t* test was used to assess paired and unpaired samples together when there was a minimum of *n* = 3 paired and unpaired samples for the comparison categories (e.g. pre-treatment vs post-treatment). Otherwise, *p* values were calculated using a paired or unpaired, two-sided, Student’s *t* test using MedCalc® Statistical Software version 20.106 (MedCalc Software Ltd, Ostend, Belgium; https://www.medcalc.org; 2022). *p* values were corrected for multiple testing using the Bonferroni method. *p* < 0.05 was considered significant for all analyses. Correlation analysis was calculated using Spearman Rho rank correlation (Medcalc v20.106). Fisher’s Exact Test was used to determine non-random associations between categorical variables. Analysis was performed on biological replicates (numbers outlined for each figure, minimum *n* = 3) or technical replicates (minimum *n* = 3) as outlined in figure legends. Grouped data are displayed as “box and whisker” plots with individual data points. The box is drawn from Quartile 1 (Q1) to Q3 with a horizontal line drawn in the middle to denote the median. The whiskers denote the distance to the highest and lowest values closest to, and inside, 1.5 times the interquartile range (Q3–Q1), starting at Q3 for the upper bar and Q1 for the lower bar. Average values +/−standard deviation (Std. Dev.) error bars are used in Fig. 6. All statistics were reviewed by the study statistician Dr. Stephen Madden.

## Results

### Patient characteristics

**Fig. 1:**
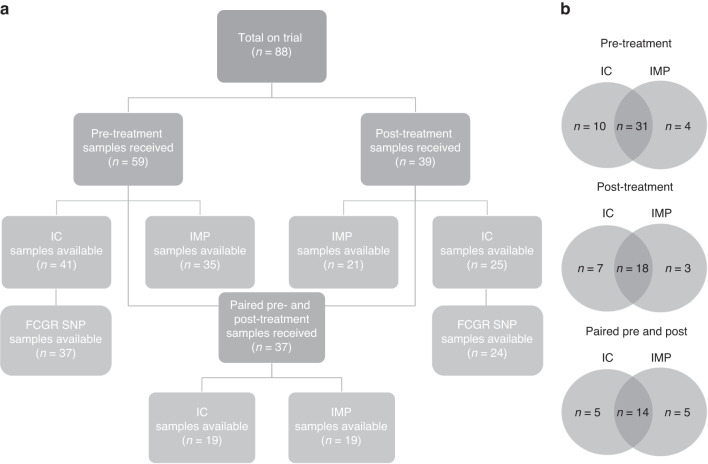
Breakdown of available samples. Consort diagram (**a**) for patient samples used in immune cytotoxicity (IC) assays, immunophenotyping (IMP) experiments and FCGR SNP genotyping. Sample overlap (**b**) between IC and IMP experiments.

Eighty-eight patients were enrolled on the TCHL study, comprising of TCH (*n* = 38), TCHL (*n* = 40) and TCL (*n* = 10) cohorts. pCR rates were not statistically different between TCH and TCHL arms, consistent with the TRIO B-07 trial (*n* = 128) that also compared TCH, TCHL and TCL [17, 22]. For this study, 47 patients in total provided a pre-treatment sample, a post-treatment sample, or both. Patient characteristics relating to the samples *analysed* are detailed in Supplementary Table 1. Figure 1a outlines the number of samples available for immune cytotoxicity (direct cytotoxicity against K562 and SKBR3/ADCC against SKBR3), immunophenotyping (CD45, CD3, CD4, CD8, CD56, CD19, CD16, CD14, PD-1) and genotyping. Samples were lost due to low post-revival viability (<70%), and the PBMC samples were prioritised for immune cytotoxicity assays. Figure 1b outlines the sample overlap between immune cytotoxicity and immunophenotyping data available within the pre-treatment, post-treatment and paired datasets. Paired pre- and post-treatment results for cytotoxicity (SKBR3–ADCC and direct) or immunophenotyping were available for 19 patients. Paired cytotoxicity data against K562 was not available for 1 of the 19 matched data sets due to a technical issue on acquisition.

### Downregulation of peripheral cytotoxic immune response following completion of neo-adjuvant chemotherapy

**Fig. 2:**
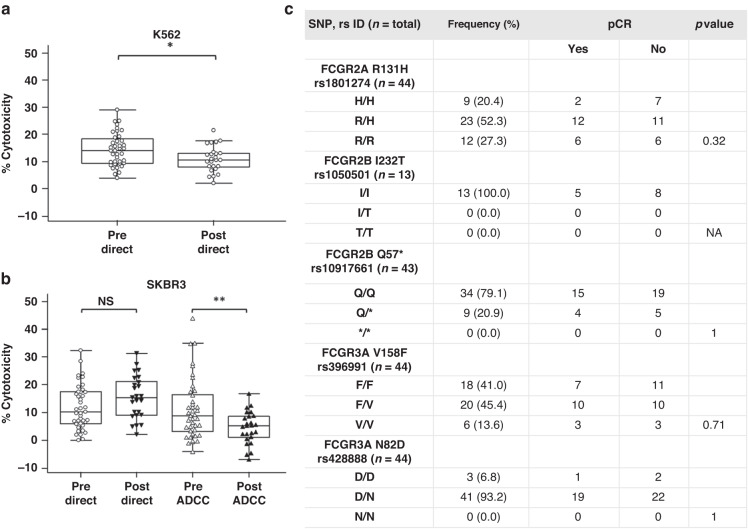
Pre- and post-treatment direct cytotoxicity, ADCC and SNP status. **a** Direct cytotoxicity levels elicited against K562 cells by patient PBMCs pre- (*n* = 40) and post-treatment (*n* = 25). The K562 dataset lost a pre-treatment sample due to a technical issue acquiring the sample. **b** Direct cytotoxicity and trastuzumab-mediated ADCC levels elicited against SKBR3 cells by patient PBMCs pre- (*n* = 41) and post-treatment (*n* = 25). An optimal pooled *t* test was used to determine statistical significance; all *p* values were adjusted for multiple testing, *p < 0.05, **p < 0.01, NS not significant. **c** FCGR SNP frequency and association with pCR in ICORG 10-05 patients. Data covers 37 pre-treatment samples and 24 post-treatment samples from (**a**) and (**b**). H histidine, R arginine, I isoleucine, T threonine, Q glutamine, D aspartic acid, N asparagine, V valine, F phenylalanine. Fisher’s Exact test, significant if *p* < 0.05, NA not available.

The cytotoxicity levels of PBMCs pre- and post-treatment were examined in all available samples. An optimal pooled *t* test allowed for paired and unpaired samples to be analysed together. PBMCs from patients who have completed neo-adjuvant chemotherapy regimens showed a significant decrease in the level of direct cytotoxicity elicited against K562 cells (*p* = 0.0133) (Fig. 2a). The same samples show no significant alteration to direct cytotoxicity against the SKBR3 cell line after treatment (*p* = 0.0954). There was a significant decrease in trastuzumab-induced ADCC mediated by the PBMCs in post-treatment samples (*p* = 0.0016) (Fig. 2b). We assessed FCGR SNP status to control for any impact these polymorphisms may have on pCR or cytotoxic capacity in this study. SNP status was not associated with pCR (Fig. 2c) or significant changes to pre-treatment ADCC levels (Supplementary Fig. 1) in this patient population. The frequency of FCGR V158F, FCGR2B I232T and FCGR2A R131H SNPs matched well with existing data [8].

### Changes to immune cell subsets following neo-adjuvant therapy

**Fig. 3:**
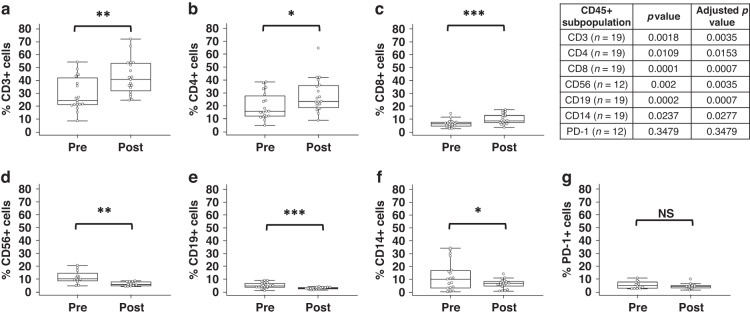
Pre- and post-treatment immune cell subsets. Proportion of CD45+ cells staining positive for **a** CD3, **b** CD4, **c** CD8, **d** CD56, **e** CD19, **f** CD14, and **g** PD-1 in paired pre- and post-treatment samples. A paired Student’s *t* test was used to determine statistical significance, all *p* values were corrected for multiple testing. *p < 0.05, **p < 0.01, ***p < 0.001, NS not significant.

**Fig. 4:**
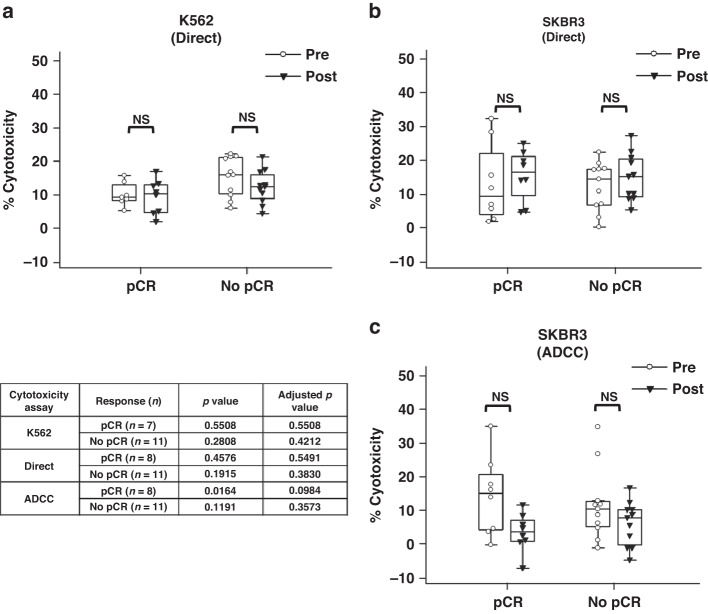
Direct cytotoxicity and ADCC by pCR status. Paired pre- and post-treatment direct cytotoxicity levels of patient PBMCs against **a** K562 and **b** SKBR3 by pCR. The K562 dataset lost a paired pCR sample due to a technical issue acquiring the sample. **c** Pre- and post-treatment trastuzumab-mediated ADCC against SKBR3 by patient PBMCs by pCR. A paired Student’s *t* test was used to determine statistical significance. All *p* values were adjusted for multiple testing. NS not significant.

**Fig. 5:**
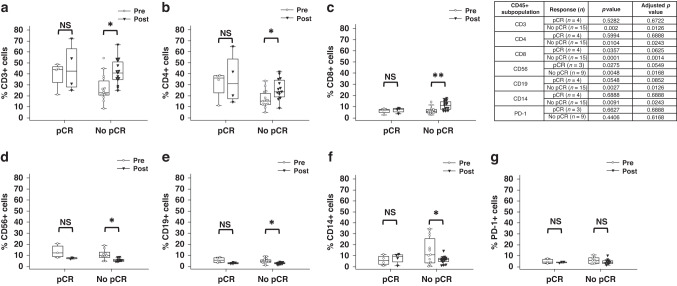
Immune cell subsets by pCR status. Proportion of CD45+ cells which stained positive for **a** CD3, **b** CD4, **c** CD8, **d** CD56, **e** CD19, **f** CD14, and **g** PD-1 in paired pre- and post-treatment samples based on pCR vs No pCR. A paired Student’s *t* test was used to determine statistical significance. All *p* values were corrected for multiple testing, *p < 0.05, **p < 0.01.

CD45+ PBMCs from patients that have completed neo-adjuvant chemotherapy regimens displayed alterations in immunophenotype. The immunophenotype data, and all following data presented in Figs. 3–5, did not meet the requirements for the use of an optimal pooled *T* test and therefore paired samples only are presented.

The proportion of CD3+ T cells significantly increased following neo-adjuvant treatment (Fig. 3a). This effect was consistent for both CD4+ and CD8+ populations (Fig. 3b, c). In pre-treatment samples, there were strong positive correlations between CD3+ and CD4+/CD8+ T cell population levels (Supplementary Fig. 2). The correlations between CD3+ and CD8+ T cells, and between CD4+ and CD8+ T cells were not present in post-treatment samples, suggesting a change in the ratio of these immune cell subsets (Supplementary Fig. 2).

Diametric changes were observed in other circulating immune cell populations. There was a significant decrease in the proportion of CD56+ NK cells (Fig. 3d), CD19+ B cells (Fig. 3e) and CD14+ monocytes (Fig. 3f) in post-treatment samples when compared with pre-treatment proportions.

Peripheral T cells are not expected to elicit cytotoxic effects in the assays used in this study as they have not been antigen-primed or otherwise activated. However, NK cells and monocytes are mediators of direct cytotoxicity against tumour cells and ADCC [41]. CD16 (FCGR3A) is a key FCGR involved in IgG1-induced ADCC and can be expressed on CD56+ NK cells and CD14+ monocytes. There was no significant alteration in the proportion of CD56+/CD16+ or CD14+/CD16+ populations between pre- and post-treatment samples (Supplementary Fig. 3). This suggests reduced in vitro trastuzumab-mediated ADCC in post-treatment samples (Fig. 2b) is unlikely to be the result of changes to CD16 expression within the NK cell and monocyte compartments. Reduced levels of CD56+ NK cells and CD14+ monocytes (Fig. 3d, f) are therefore the most likely reason for the reduced direct cytotoxicity against K562 (Fig. 2a) and trastuzumab-mediated ADCC against the SKBR3 cell line (Fig. 2b).

Expression of the immune checkpoint PD-1 was investigated in CD45+ PBMCs to assess potential immunosuppressive phenotypes. CD45+PD-1+ immune cell levels were unchanged between pre- and post-treatment samples (Fig. 3g).

### Post-treatment cytotoxic immune response did not vary based on pCR and no pCR

To ascertain if variances in in vitro PBMC cytotoxicity due to neo-adjuvant treatment could be associated with patient response to therapy, the in vitro cytotoxicity data was broken down by pCR. There was no change in direct cytotoxicity against the K562 or SKBR3 cell lines based on clinical response between pre- and post-treatment samples (Fig. 4a, b). A post-treatment decrease in trastuzumab-mediated ADCC against the SKBR3 cell line for the pCR cohort did not pass correction for multiple testing (Fig. 4c).

### Post-treatment changes in immune cell subsets are driven by the No pCR cohort

Immunophenotype data was also assessed in the context of pCR. There were no significant differences in the immune cell populations pre- and post-treatment within the pCR cohort (Fig. 5a–f). In contrast, the No pCR cohort reported significant post-treatment differences in CD3+, CD4+, CD8+, CD56+, CD14+, and CD19+ immune cells (Fig. 5a–f). CD3+, CD4+, and CD8+ immune cell subsets were significantly higher post-treatment in the No pCR group (Fig. 5a–c). CD56+ NK cell, CD14+ monocyte and CD19+ B cell populations displayed significantly reduced post-treatment levels in the No pCR cohort (Fig. 5d–f). Pre- and post-treatment CD45+PD-1+ cells did not differ within pCR and No pCR cohorts (Fig. 5g). This data suggests that the overall treatment associated changes in immune cell proportions reported in Fig. 3 are predominantly driven by the post-treatment No pCR cohort.

### Baseline circulating immune cells from patients with no pCR have augmented cytotoxic response after treatment with pembrolizumab

**Fig. 6:**
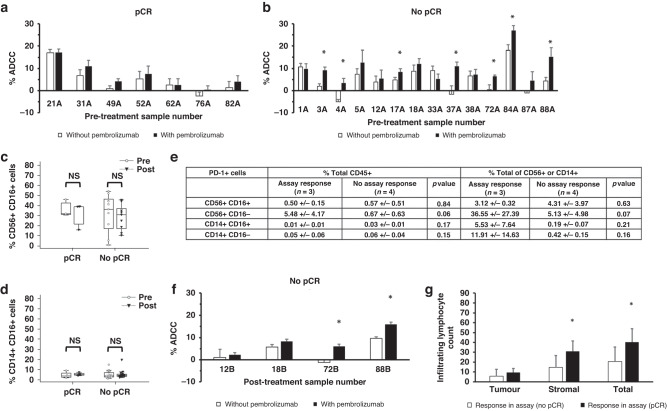
Pembrolizumab-responsive PBMCs in the No pCR cohort. Trastuzumab ADCC levels with and without pembrolizumab against SKBR3 cells using pre-treatment patient PBMCs from **a** pCR (*n* = 7) and **b** No pCR (*n* = 14) cohorts. Bars represent the average of three technical replicates +/− Std. Dev. **c** Comparison of pre- and post-treatment CD56+CD16+ levels by treatment response. **d** Comparison of pre- and post-treatment CD14+CD16+ levels by treatment response. **e** PD-1+CD56 +CD16+/− and PD-1+CD14+CD16+/− as a percentage of CD45+ immune cells or subset total (CD56+ or CD14+) +/− Std. Dev. based on assay response in (**a**) and (**b**). **f** Trastuzumab ADCC levels with and without pembrolizumab against SKBR3 cells using post-treatment patient PBMCs from the No pCR patient cohort. Average of three technical replicates +/− Std. Dev. **g** Average pre-treatment infiltrating lymphocyte levels in tumour, stroma and combined for patients whose pre-treatment PBMC samples responded to pembrolizumab in (**b**) (*n* = 6) versus patient samples that did not respond to pembrolizumab in (**a**) (*n* = 7) +/− Std. Dev. Unpaired or paired (**c**, **d**) Student’s *t* tests were used to determine statistical significance, *p < 0.05.

Examining PD-1 expression on CD56+, CD8+, and CD14+ subsets reveals that PD-1 expression was present but there was no difference between pre- and post-treatment when divided based on treatment response (Supplementary Fig. 3). NK cells are subject to PD-1-mediated immune suppression [42]. To investigate whether PD-1-mediated immunosuppression of in vitro cytotoxic function could be playing a role in pCR, the anti-PD-1 inhibitor pembrolizumab was added to ADCC assays for any remaining pre-treatment PBMC samples (*n* = 21). Pembrolizumab did not increase the direct cytotoxicity elicited by patient PBMCs against SKBR3 or K562, regardless of treatment outcome (Supplementary Fig. 4). In all, 0/7 pCR samples displayed an increase in trastuzumab-mediated ADCC with the addition of pembrolizumab (Fig. 6a). There was a significant increase in trastuzumab-mediated ADCC with the addition of pembrolizumab in 7/14 patients who had No pCR status (Fig. 6b). No significant differences in CD56+CD16+ and CD14+CD16+ proportions were identified within the pCR and no pCR cohorts overall (Fig. 6c, d). A limited number of the PBMC samples in Fig. 6a, b had matched immunophenotype data. Examination of PD-1-expressing CD56+CD16+/CD16− and CD14+CD16+/CD16− subsets as % of total CD45+ cells or parental CD56+ and CD14+ subsets did not reveal any significant differences between those samples that responded to pembrolizumab in vitro and those that did not (Fig. 6e). Although not reaching statistical significance, an 8.2-fold higher level of PD-1+CD56+CD16− cells was observed in PBMC samples that responded to pembrolizumab in the assay, compared to those PBMC samples that did not respond (Fig. 6e).

In addition, the pre-treatment PD-1 inhibited ADCC response (Fig. 6b) was shown to be durable, being detected in two post-treatment samples 18 weeks (six rounds of therapy) after the pre-treatment samples were taken (Fig. 6f). Analysis of the pembrolizumab-sensitive samples based on tumour oestrogen receptor (ER) and progesterone receptor (PR) expression revealed 7/7 were associated with ER+ tumours and 5/7 were associated with ER+/PR+ tumours (Supplementary Fig. 5). This compared to 2/7 ER+ (0/7 ER+/PR+) for the pembrolizumab-insensitive pCR samples (Supplementary Fig. 5).

### TIL levels are lower in patients with in vitro PBMC response to pembrolizumab

Matched pre-treatment TIL data were available for 13/21 pre-treatment PBMC samples utilised in the pembrolizumab ADCC assays [40]. Tumours from patients with a pembrolizumab-responsive ADCC response (*n* = 6, No pCR) had significantly lower levels of stromal infiltrating lymphocytes (*p* = 0.025), and combined tumour and stromal infiltrating lymphocytes (*p* = 0.029), compared to tumours from patients with a pCR who showed no significant alteration to ADCC levels with the addition of pembrolizumab (*n* = 7) (Fig. 6g).

### Treatment arm did not affect immune cytotoxicity or PBMC immunophenotype

No significant difference was found when post-treatment direct cytotoxicity (K562/SKBR3) or ADCC (SKBR3) were compared to pre-treatment levels within arms (Supplementary Fig. 6) or divided by pCR within treatment arms (Supplementary Fig. 7). Treatment arm did not affect post-treatment changes to CD3+, CD4+, CD8+, CD56+, CD19+, CD14+, and PD-1+ immune cell subpopulations (Supplementary Fig. 8). The increase in CD8+ T cells verged on significance for the TCHL arm only (*p* = 0.05), as did a decrease in CD19+ B cells in TCH and TCHL arms only (*p* = 0.05).

## Discussion

We report that neo-adjuvant treatment reduced the direct cytotoxic and ADCC capacity of circulating immune cells for all patients on the ICORG 10-05 trial, irrespective of FCGR SNP status. This corresponded with post-treatment increases in circulating CD8+ and CD4+ T cells, and decreased levels of NK cells, monocytes and B cells. When assessed in the context of treatment response, these changes were predominantly associated with patients who did not achieve a pCR. In addition, functionally pembrolizumab-responsive ADCC-capable immune cells were only detected in the pre-therapy blood of patients who did not achieve a pCR, had lower TIL levels and were more likely to have ER+ tumours.

This study is the first to genotype, phenotype and functionally assess patient PBMCs in vitro in the context of pCR following 6 rounds of neo-adjuvant anti-HER2 targeted therapy/docetaxel/carboplatin. We found no statistically significant evidence that the HER2-targeted therapy given influenced the in vitro cytotoxicity or immune profile of samples (Supplementary Figs. 6–8), therefore the potential impact of the chemotherapy backbone (docetaxel/carboplatin) received by all patients must be considered. Chemotherapy can stimulate the immune response through release of antigens from chemotherapy-induced cell lysis [43]. Taxane-based regimens can be beneficial in reducing immunosuppressive cell populations, such as myeloid-derived suppressor cells and regulatory T cells [44, 45]. In vitro studies have also provided evidence that carboplatin can directly enhance ADCC [46]. These data highlight the importance of the chemotherapy backbone to the immune response in HER2+ breast cancer, and the need for additional work in this area in order to optimise the anti-tumour immune response, particularly in conjunction with therapeutic antibodies.

Previous studies, such as those from Gennari et al. and Varchetta et al., have shown an increase in trastuzumab-mediated ADCC capabilities of patient immune cells in vitro after receiving neo-adjuvant trastuzumab monotherapy [10, 11]. Beano et al. demonstrated that the PBMCs from patients they classified as clinical responders had higher in vitro trastuzumab-mediated ADCC and direct cytotoxicity levels following one round of maintenance trastuzumab treatment compared to non-responders [9]. It has been reported in a limited patient cohort that the number of circulating NK cells can be reduced following neo-adjuvant trastuzumab/docetaxel treatment [47]. This is in line with the reduced post-treatment ADCC and direct cytotoxicity (Fig. 2), and reduced CD56+ and CD14+ immune subsets in peripheral blood in this study (Fig. 3). Interestingly, circulating NK cell levels decrease following six cycles of treatment in the No pCR cohort only, and gene signature analysis of a small cohort of ICORG 10-05 patients showed that activated tumour NK cell levels increased following one cycle [34]. A study comparing circulating NK cells and tumour infiltration levels in patients at the same post-treatment time point would be required to provide data on this potential inverse correlation. When our matched pre- and post-treatment in vitro cytotoxicity data was stratified by pCR, there was no significant difference in direct cytotoxicity or ADCC within pCR and No pCR groups, although additional work is required to confirm if higher pre-treatment ADCC may be associated with pCR in a larger dataset (Fig. 4). The FCGR polymorphisms we examined are involved in ADCC and have been associated with higher affinity for IgG Fc (FGR2A 131H>FGR2A 131R, FCGR3A 158V>FCGR3A 158F), impaired negative regulatory activity (FCGR2B 232T vs FCGR2B 232I), a non-functional truncated form (FCGR2B Q57*) and higher FCGR3A mRNA expression levels (FCGR3A D/N vs FCGR3A D/D) [8, 48, 49]. FCGR3A 158V carriers and FCGR2A 131H homozygotes have been shown to associate with pCR to trastuzumab in the neo-adjuvant setting [8]. In this study, the polymorphisms examined did not significantly associate with pCR (Fig. 2c) or ex vivo pre-treatment PBMC ADCC levels (Supplementary Fig. 1) but the results suggest that larger sample numbers will be needed to investigate this fully.

The increase in post-therapy T cell levels indicates a triggering of an adaptive immune response. When assessed by pCR, it was found that the No pCR cohort was making the biggest contribution to the observed post-treatment changes for all immune cell subtypes (Fig. 5). This suggests an ongoing and unresolved anti-tumour adaptive immune response in the No pCR cohort. The proportion of circulating PD-1+ immune cells was not impacted by treatment (Fig. 3) or associated with treatment response (Fig. 5). However, it was found that baseline circulating ADCC-capable immune cells that are functionally suppressed by PD-1 are potentially indicative of HER2+ breast cancer patients that will not achieve optimal benefit from neo-adjuvant treatment (Fig. 6b). As this in vitro response can be measured on a patient-by-patient basis, it has potential as a biomarker of response to standard therapy, capable of stratifying patients for clinical escalation/de-escalation studies. When compared to tumour biopsies, blood-based biomarkers represent a more readily accessible option with potential for monitoring of response. Recent advances have also meant that peripheral blood cells are being characterised in unprecedented resolution, improving their potential as biomarkers [50]. The T Cell Receptor (TCR) repertoire diversity of peripheral PD-1+CD8+ T cells is reported as a predictive biomarker of response to immunotherapy, for example [51]. Here, the flow cytometry-based assay determined target cell death on a cell-by-cell basis, providing greater sensitivity than methods used in past studies [9–11, 37]. The endurance of this in vitro response following 18 weeks of therapy is supportive of a robust potential biomarker (Fig. 6f). The high prevalence of ER+/PR+ tumours associated with the presence of pembrolizumab-responsive ADCC-capable immune cells (Supplementary Fig. 5) warrants further investigation, especially given recent interest in assigning “triple positive” breast cancer (ER+/PR+/HER2+) as a distinct subtype [52].

Our results highlight functional assessment of immune cells as an important adjunct to phenotypic analysis, which in this case did not definitively identify PD-1+CD16+ NK cells or monocytes as markers of treatment response in the population as a whole (Fig. 6c, d). CD56+ NK cells are the prime candidates for mediating the response to pembrolizumab in vitro, with PD-1+CD56+ NK cells comprising a 19 to 50-fold higher proportion of the CD45+ population than PD-1+CD14+ monocytes (Fig. 6e). Traditionally, CD16+ NK cells have been characterised as the main mediators of ADCC within the PBMC compartment and reports suggest that PD-1 expression is predominantly associated with CD16+ NK cell populations [41, 42]. The limited data in this study suggests that PD-1+CD56+CD16− cells are also worthy of further investigation.

The reduced TIL count in tumours from patients with PBMCs that responded to pembrolizumab in vitro is an important indication that the circulating immune cells are associated with an immune-suppressed tumour microenvironment (Fig. 6g). A meta-analysis of 5 trials (CHER-LOB, GeparQuattro, GeparQuinto, GeparSixto and Neo-ALTTO) involving neo-adjuvant treatment of HER2+ breast cancer with trastuzumab and chemotherapy (*n* = 1256) showed a significant correlation between high baseline TILs and achieving a pCR [53]. This association between higher TIL levels and pCR has been confirmed in ICORG 10-05, with <5% residual tumour and reduced tumour-associated CD4+ and CD8+ T cells after one cycle of therapy in the pCR cohort [40]. The opposite has been reported for many patients with extensive residual disease following neo-adjuvant therapy, with significant but dysfunctional immune presence in the tumour microenvironment [54, 55]. These data and previous work from our lab [34], suggest that the majority of patients having a pCR have a resolved anti-tumour immune response at a very early phase. In contrast, those with No pCR display signs of an ongoing, but ineffective, adaptive immune response (Fig. 5), associated with impaired TIL levels (Fig. 6g) and PD-1-mediated immunosuppression at baseline (Fig. 6b).

It should also be noted that the presence of PD-1-inhibited immune cells could also identify patients who will benefit from the addition of anti-PD-1 immune checkpoint inhibitors in the neo-adjuvant setting. Clinical trials examining checkpoint inhibitors in breast cancer are established, with anti-PD-L1 therapy atezolizumab approved to treat TNBC following the IMpassion130 trial [56]. However, the IMpassion050 trial reported no advantage for the addition of atezolizumab to neo-adjuvant trastuzumab/pertuzumab/chemotherapy in PD-L1+HER2+ breast cancer [57]. The PANACEA trial showed 15% of PD-L1+ patients displaying objective responses to pembrolizumab in HER2+ breast cancer patients who were resistant to trastuzumab [58]. This suggests further exploration of checkpoint inhibitors in TNBC, HER2+ and ER+ breast cancer subtypes, in both therapy-naive and therapy-refractory settings, should be undertaken to compare anti-PD-1 and anti-PD-L1 therapies for differential therapeutic efficacy, and assess if the biomarker described in this manuscript has capacity to identify checkpoint inhibitor responsive patients, alone or in combination with existing biomarkers.

In summary, we hypothesise that circulating anti-PD-1-sensitive ADCC-capable immune cells identify immune-suppressed tumour phenotypes and could function as a biomarker of response to standard chemotherapy regimens and immune checkpoint inhibitors. Current efforts are focussed on prospective collection of pre-treatment blood samples from early stage HER2+ breast cancer patients and non-small cell lung cancer and melanoma patients scheduled to receive immune checkpoint therapy to provide larger datasets to assess the potential of the functional biomarker assay described in this study.

## Supplementary information

Supplementary Table 1 and Supplementary Figures 1-8

Supplementary detail on direct cytotoxicity and ADCC assays

Minimum information about a flow cytometry experiment

## Data Availability

The datasets generated during and/or analysed during the current study are not publicly available due to the terms of ethics approval/consent for the study and data privacy regulations but are available from the corresponding author on reasonable request.
