# Supplementary material for: Alterations in immune cell phenotype and cytotoxic capacity in HER2+ breast cancer patients receiving HER2-targeted neo-adjuvant therapy
Source: Br J Cancer. 2023 Jul 28;129(6):1022–31. doi: 10.1038/s41416-023-02375-y (PMC10491671; doi:10.1038/s41416-023-02375-y)
Supplement: Supplementary file 1 — Supplementary Table 1 and Supplementary Figures 1-8 [file 41416_2023_2375_MOESM1_ESM.pdf]

Gaynor et al.
Supplementary Table 1 and Supplementary Figures 1-8

Supplementary Table 1: Characteristics of ICORG 10-05 patients (n=47) included in this study.

	n	%
**Total**	47	100
**Age**		
Median (years)	48	
Range (years)	34-80	
**Trial Arm**		
TCHL	23	48.9
TCH	16	34.1
TCL	8	17.0
**ER status**		
Negative	17	36.2
Positive	30	63.8
**PR status**		
Negative	27	57.4
Positive	20	42.6
**Treatment outcome**		
pCR	22	46.8
No pCR	25	53.2

TC – docetaxel/carboplatin, H – trastuzumab, L – lapatinib, ER- estrogen receptor, PR-progesterone receptor, pCR – pathological complete response.

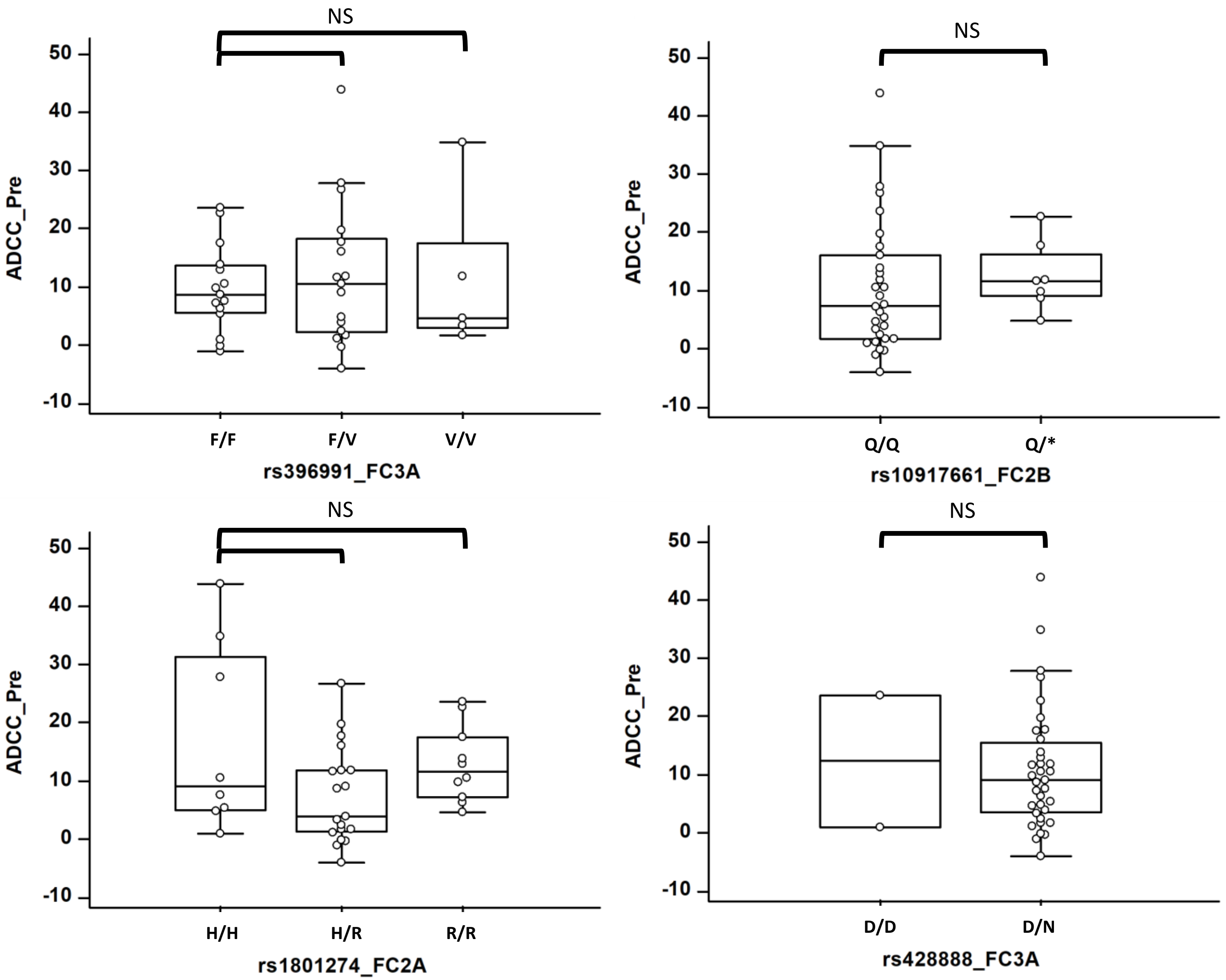

Supplementary Figure 1. Pre-treatment *in vitro* trastuzumab-mediated ADCC levels induced by PBMCs from ICORG 10-05 patients against SKBR3 cells based on SNP status. F= phenylalanine, V = valine, Q = glutamine, H= histadine, R =arginine, D= aspartic acid , N= asparagine. No significant difference in ADCC levels was detected based on SNP status. rs1050501 was not included as only reference alleles were reported. Independent Student's T test. * $p < 0.05$, NS designates tests that were Not Significant without multiple testing.

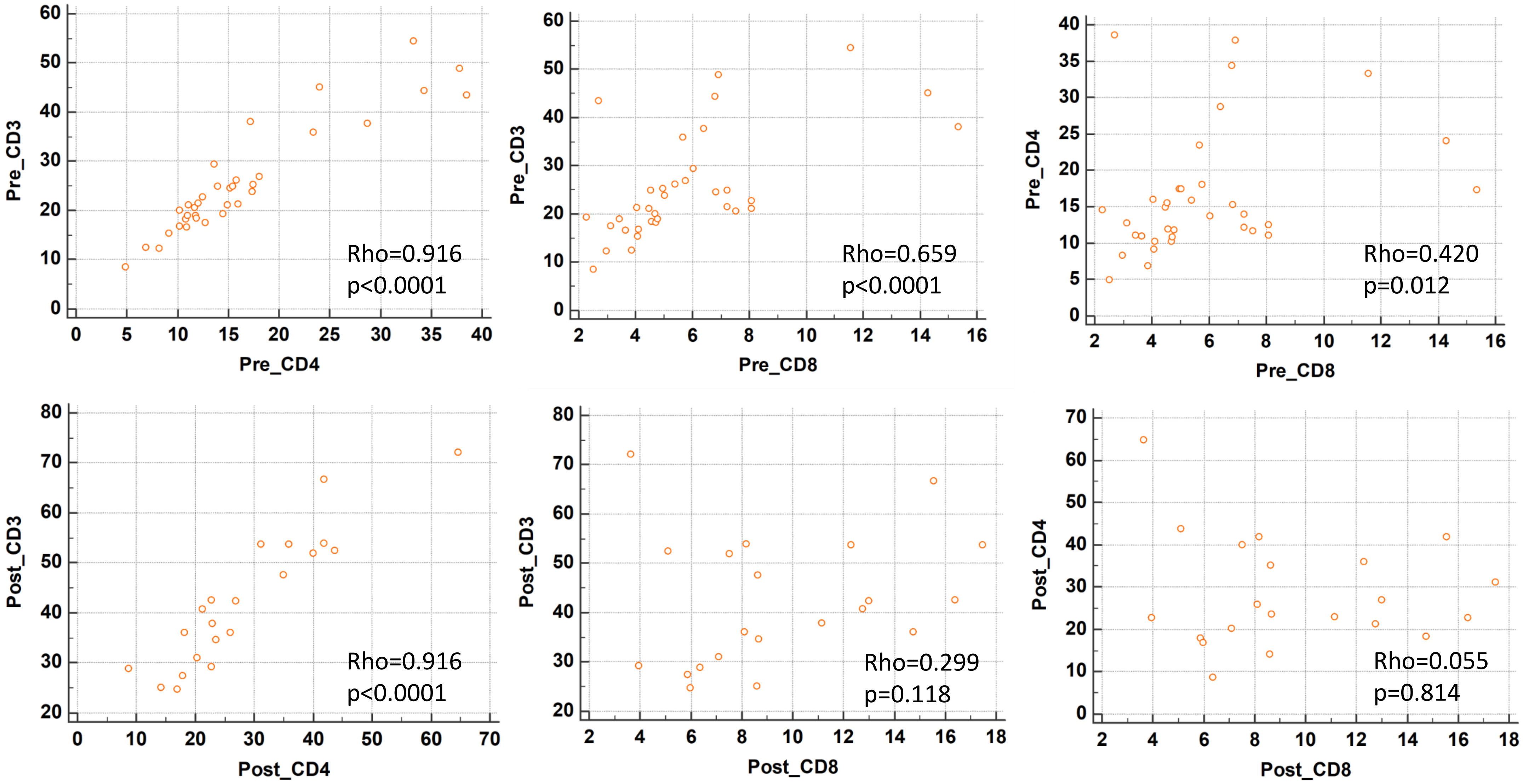

Supplementary Figure 2: Spearman's rho ranked correlation test for CD3+, CD4+ and CD8+ T cell populations in pre-treatment (n=35) and post-treatment (n=21) samples.

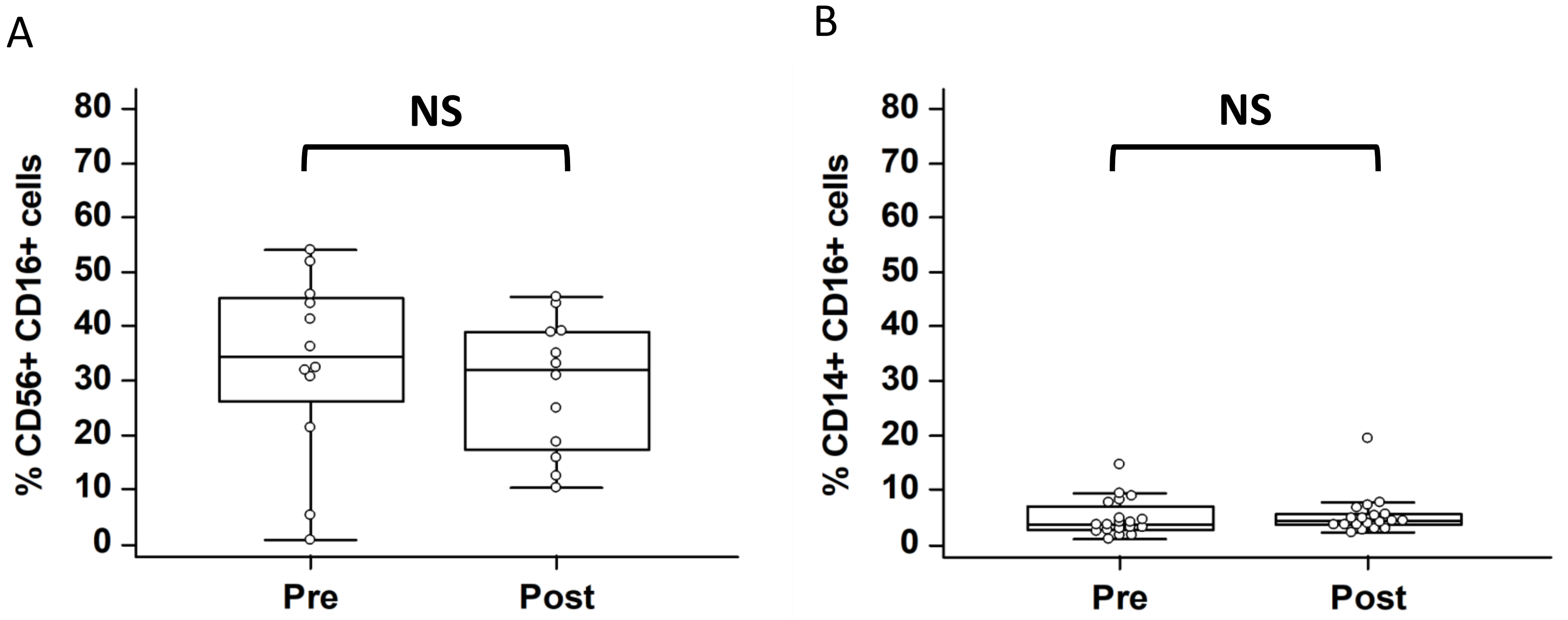

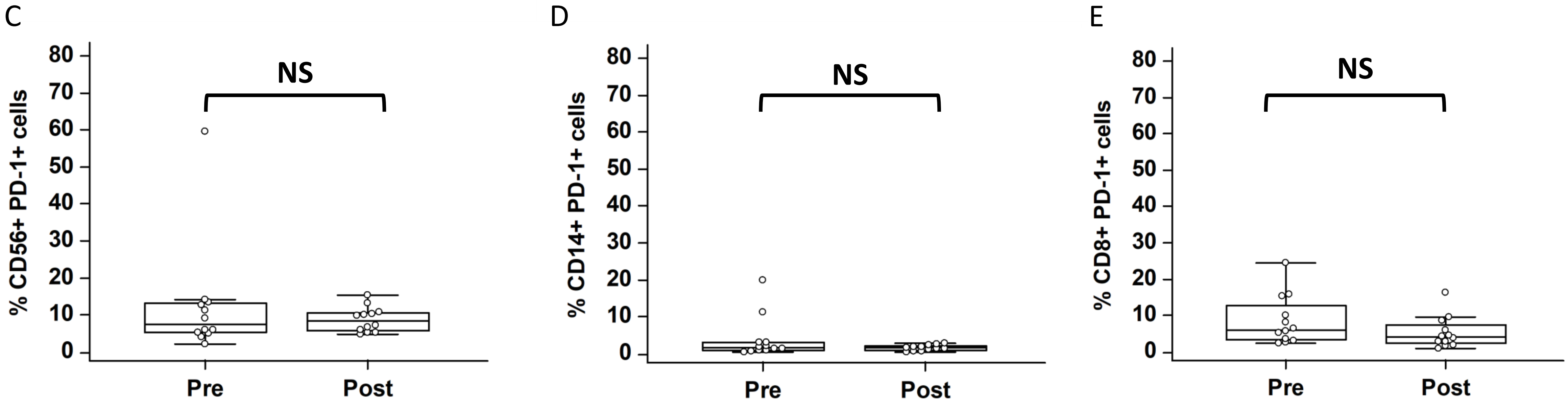

Supplementary Figure 3. Proportion of CD45+ cells staining positive for A) CD56/CD16, B) CD14/CD16, C) CD56/PD-1, D) CD14/PD-1, E) CD8/PD-1 in paired pre-treatment and post-treatment samples. A paired student's t test was used to determine statistical significance, all p values were corrected for multiple testing * $p < 0.05$, ** $p<0.01$, *** $p<0.001$, NS not significant.

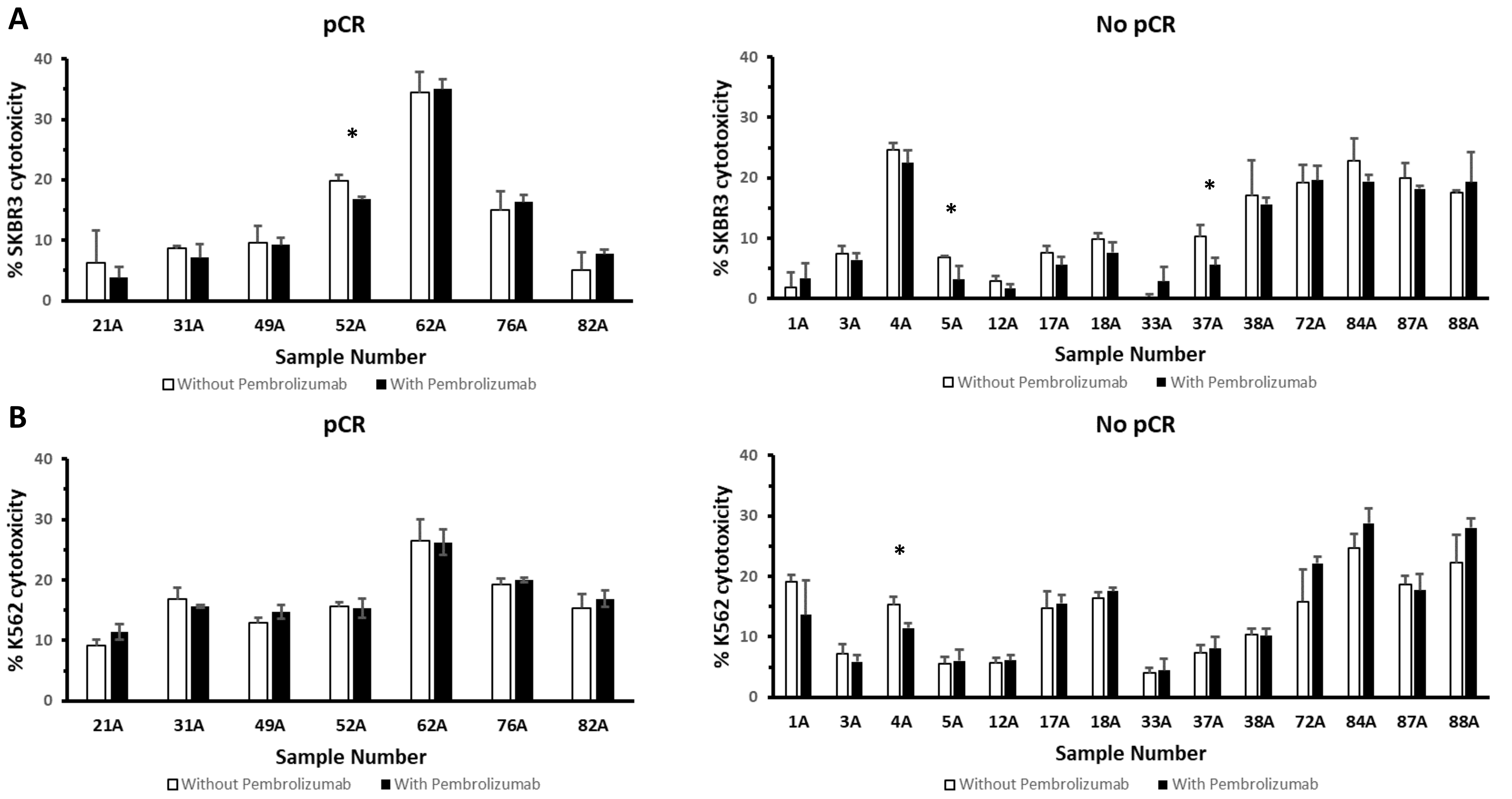

Supplementary Figure 4 A) Direct cytotoxicity levels elicited against SKBR3 cells by pre-treatment patient PBMCs from pCR (n=7) and No-pCR (n=14) cohorts with and without pembrolizumab. Effector cells to target cell ratio of 10:1. B) Direct cytotoxicity levels elicited against K562 cells by pre-treatment patient PBMCs from pCR (n=7) and No-pCR (n=14) cohorts with and without pembrolizumab. Effector cells to target cell ratio of 10:1. A paired Student's T test was used to determine statistical significance. * $p < 0.05$

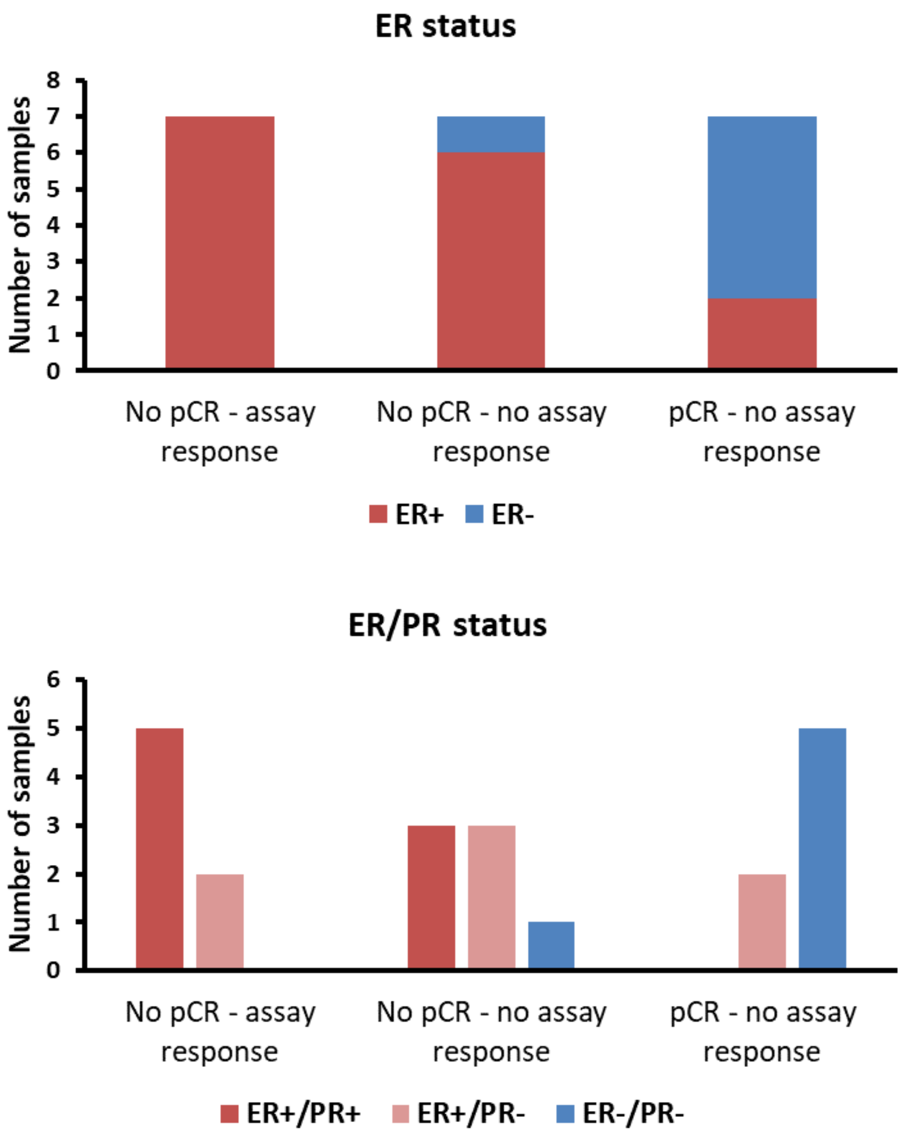

Supplementary Figure 5: Breakdown of ER+ **(A)** and ER+/PR+ **(B)** status of patient samples (n=21) by response in pembrolizumab-ADCC assay/pCR status (Main Figure 6A).

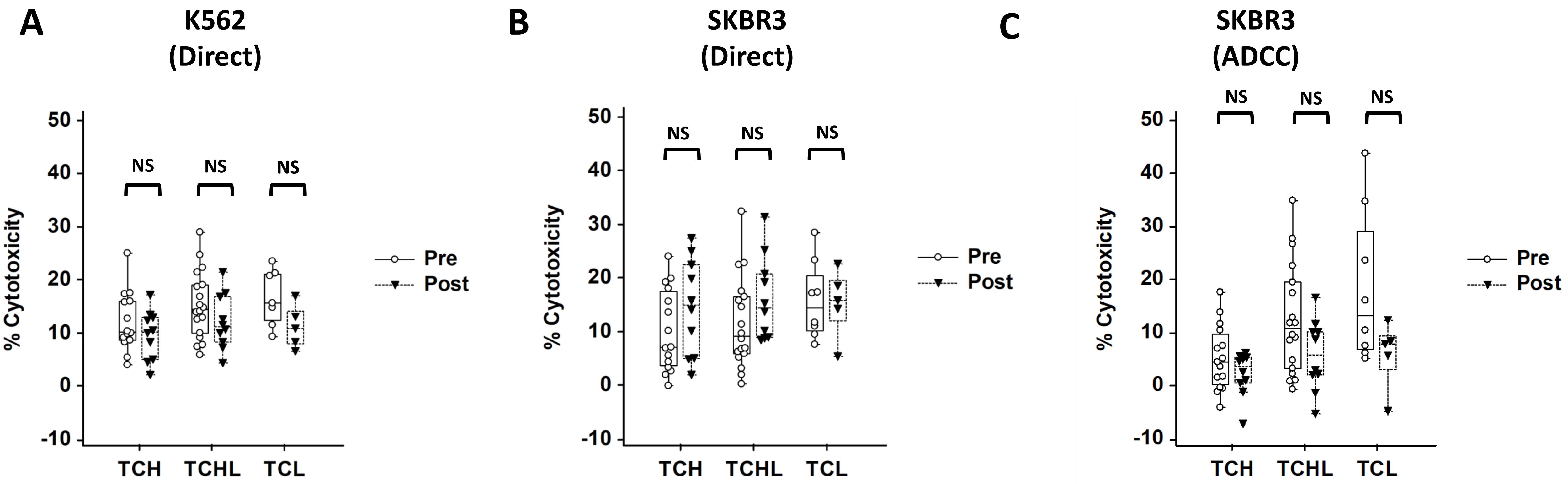

Arm	Assay	Paired Sample (n)	p value	Adjusted p value
TCH	K562	7	0.25	0.55
	Direct (SKBR3)	7	0.03	0.23
	ADCC	7	0.12	0.51
TCHL	K562	7	0.86	0.93
	Direct (SKBR3)	7	0.58	0.87
	ADCC	7	0.20	0.55
TCL	pCR	6	0.72	0.92
	No pCR	15		
	pCR	6	0.37	0.67
	No pCR	15		
PD-1	pCR	4	0.93	0.93
	No pCR	9		

Supplementary Figure 6. Comparison of direct **(A, B)** and antibody-dependent **(C)** cytotoxicity levels of pre- and post-treatment patient PBMCs by treatment arm. TCH, n= 15, TCHL, n= 18, TCL, n= 8. Paired Student’s T test. * $p < 0.05$, NS designates tests that were Not Significant.

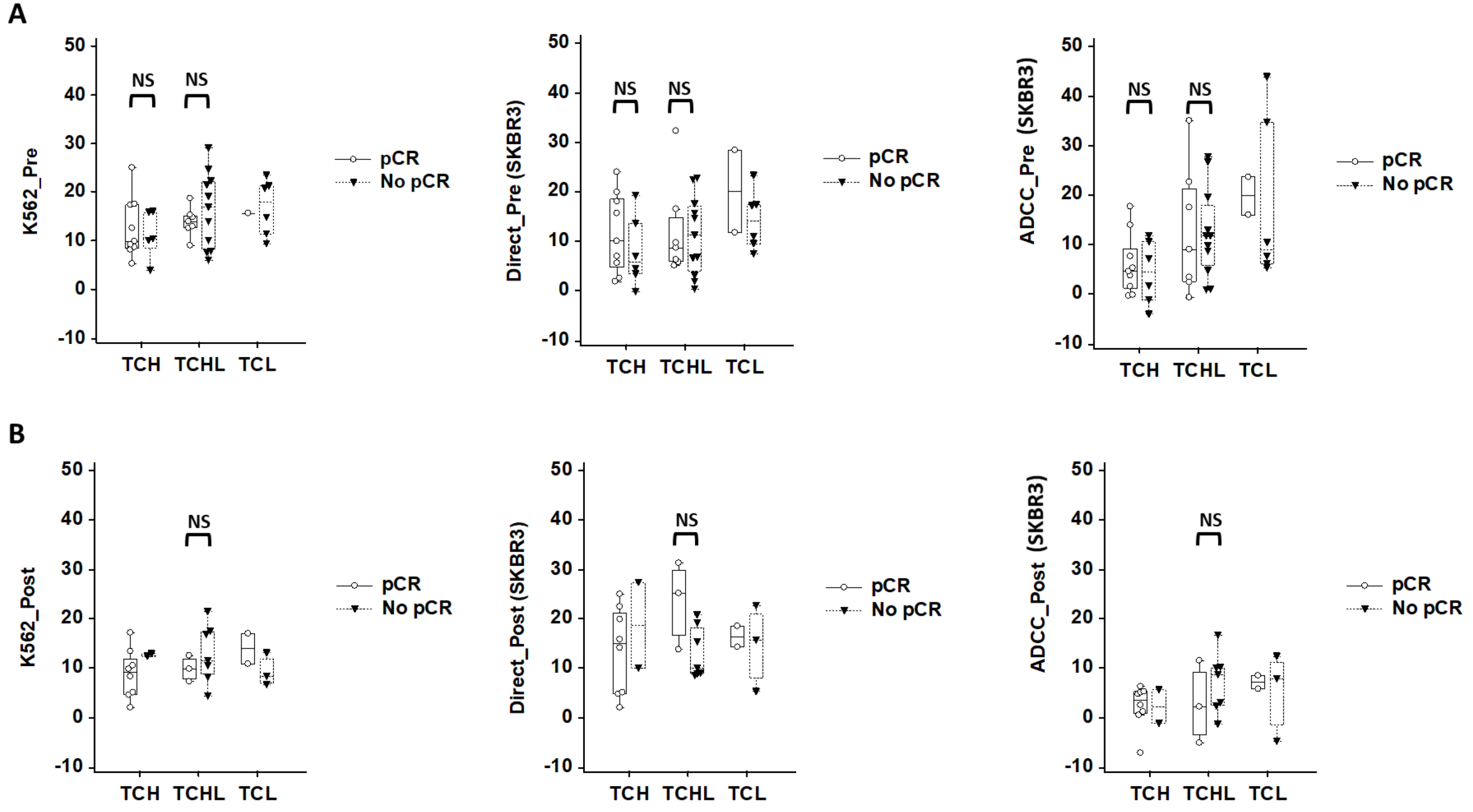

Arm	Assay	Pre or Post	pCR (n)	No pCR (n)	p value	Adjusted p value
TCH	K562	Pre	9	5	0.67	0.97
		Post	8	2	0.33	0.96
	Direct (SKBR3)	Pre	9	6	0.38	0.96
		Post	8	2	0.51	0.96
	ADCC	Pre	9	6	0.62	0.97
		Post	8	2	0.97	0.97
TCHL	K562	Pre	7	11	0.47	0.96
		Post	3	7	0.43	0.96
	Direct (SKBR3)	Pre	7	11	0.84	0.97
		Post	3	7	0.05	0.80
	ADCC	Pre	7	11	0.94	0.97
		Post	3	7	0.39	0.96
TCL	K562	Pre	1	6	n/a	n/a
		Post	2	3	0.27	0.96
	Direct (SKBR3)	Pre	2	6	0.37	0.96
		Post	2	3	0.80	0.97
	ADCC	Pre	2	6	0.89	0.97
		Post	2	3	0.79	0.97

Supplementary Figure 7. Comparison of direct (K562 and SKBR3)) and antibody-dependent (SKBR3) cytotoxicity levels for pCR and No pCR patient PBMCs by treatment arm for both pre- **(A)** and post **(B)**-treatment samples. Pre-sample numbers – TCH, n= 15, TCHL, n= 18, TCL, n= 8. Post-sample numbers TCH, n= 10, TCHL, n= 10, TCL, n= 5. An unpaired Student's T test was used to determine statistical significance for pCR vs No pCR samples in groups with a minimum of n=3. No comparison provided a statistically significant difference ($p < 0.05$), with those groups tested marked as NS (not significant).

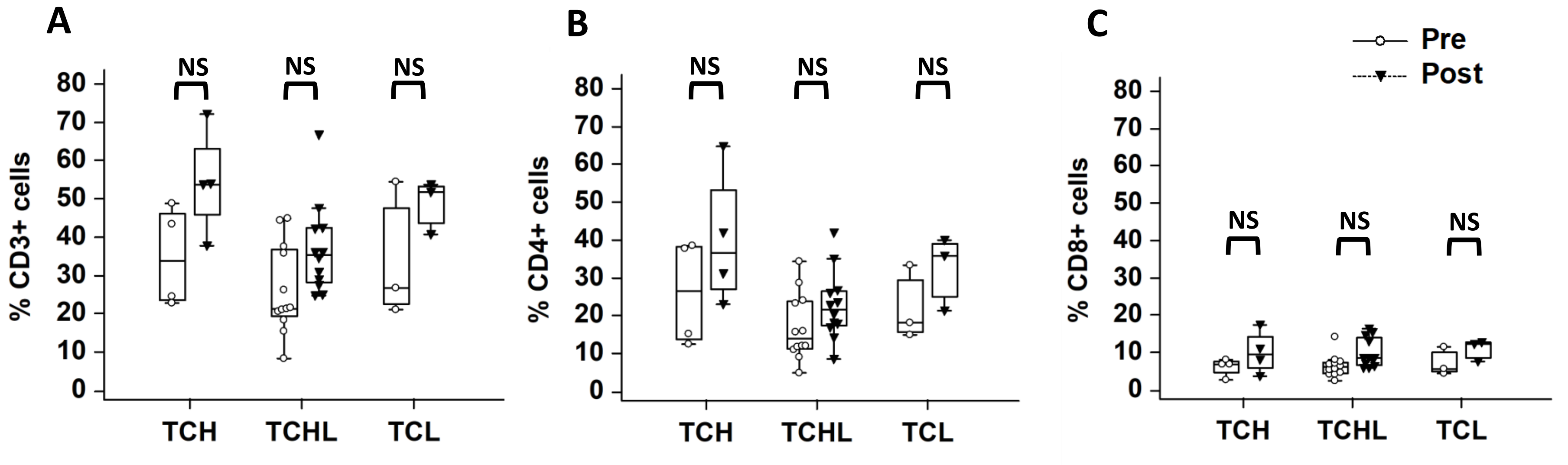

CD45+ population	Arm	Paired Sample (n)	p value	Adjusted p value
CD3	TCH	4	0.05	0.18
	TCHL	12	0.03	0.16
	TCL	3	0.41	0.49
CD4	TCH	4	0.07	0.20
	TCHL	12	0.12	0.25
	TCL	3	0.46	0.51
CD8	TCH	4	0.14	0.25
	TCHL	12	3.60x10-3	0.05
	TCL	3	0.15	0.25
CD56	TCH	3	0.19	0.27
	TCHL	6	0.04	0.16
	TCL	3	0.15	0.25
CD19	TCH	4	9.30x10-3	0.07
	TCHL	12	5.00x10-3	0.05
	TCL	3	0.09	0.21
CD14	TCH	4	0.08	0.21
	TCHL	12	0.19	0.21
	TCL	3	0.42	0.49
PD-1	TCH	3	0.69	0.73
	TCHL	6	0.78	0.73
	TCL	3	0.42	0.49

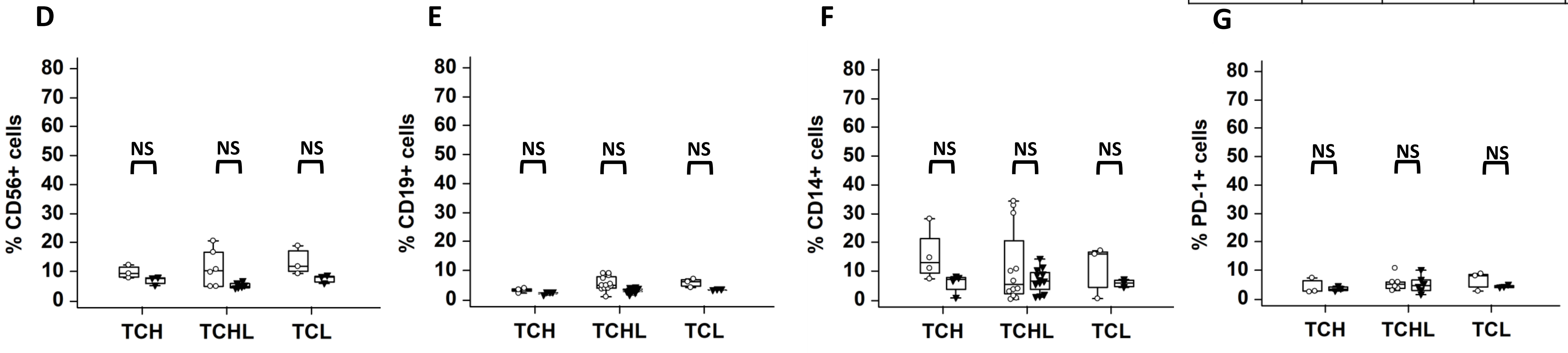

Supplementary Figure 8. Percentage of CD45+ cells staining positive for A) CD3, B) CD4, C) CD8, D) CD56†, E) CD19, F) CD14, and G) PD-1† in paired TCH (n=4), TCHL (n= 12) and TCL (n=3) samples. A paired student's t test was used to determine statistical significance, all p values were corrected for multiple testing, * $p < 0.05$. † TCH (n=3), TCHL (n=6) and TCHL (n=3)
