# Supplementary material for: Alterations in immune cell phenotype and cytotoxic capacity in HER2+ breast cancer patients receiving HER2-targeted neo-adjuvant therapy
Source: Br J Cancer. 2023 Jul 28;129(6):1022–31. doi: 10.1038/s41416-023-02375-y (PMC10491671; doi:10.1038/s41416-023-02375-y)
Supplement: Supplementary file 2 — Supplementary detail on direct cytotoxicity and ADCC assays [file 41416_2023_2375_MOESM2_ESM.docx]

**Direct cytotoxicity assay and ADCC assay protocol – Gaynor et al.**

SKBR3 and K562 target cells (TCs) were seeded 48 hours prior to assay commencement to achieve 70-80 % confluence on the day of the experiment. TCs were stained with 0.1 mM carboxyfluorescein succinimidyl ester (CFSE) (Sigma Aldrich 21888). Adherent cells were detached using <5 minute exposure to trypsin/EDTA. TC number and viability was determined using a flow cytometry-based method (Luminex Viacount). TCs and PBMC effector cells (ECs) were then normalized to 1.0-2.5 x 10^5^ cells/mL, depending on EC cell viability. Due to an inverse correlation between cytotoxicity and viability, EC samples with a viability of less than 70% were excluded from immune cytotoxicity analysis in line with previous studies. Post-treatment TCL arm samples were utilised as a control for post-treatment TCH/TCHL arm samples to ensure there was no residual trastuzumab contamination present.

TCs and ECs were aliquoted and trastuzumab 10 µg/mL and/or pembrolizumab 10 µg/mL added. Cells were plated in 96 well round bottom plates in triplicate wells at a ratio of 10:1 (EC: TC) in a volume of 200 µL. Control wells included basal cell death +/- trastuzumab and/or pembrolizumab, 100 % TC dead cell controls, ECs only and a negative control for ADCC (CD20-specific rituximab, 10 µg/mL). The plates were centrifuged at 50G for 3 minutes and incubated for 4 hours at 37^0^C. To stop the assay, 40 µL aminoactinomycin-D (7AAD) (Sigma Aldrich 9400) was added to each well and mixed gently to disrupt EC:TC interactions. The plates were read on a Guava Easycyte flow cytometer using the Guava InCyte program (Luminex) to determine the % viability of the TC population.

**Calculation of direct cytotoxicity and ADCC values – Gaynor et al.**

**ADCC= antibody-dependent cell-mediated cytotoxicity**

**PBMC= Peripheral blood mononuclear cell**

**EC = effector cell**

**TC = target cell**

**Direct cytotoxicity and trastuzumab-mediated ADCC**

All values referred to below were determined from the flow cytometry experiment as a % of the TC population within each stated condition.

Direct PBMC-mediated cytotoxicity (EC Cytox) was calculated by subtracting basal TC death from TC death in the presence of ECs only for K562 and SKBR3.

% direct cytotoxicity (EC Cytox) = (EC: TC, 10:1) – (EC:TC, 0:1 control)

In SKBR3, basal TC death in the presence of trastuzumab (EC:TC, 0:1 + trastuzumab control) was subtracted from (EC: TC, 10:1 + trastuzumab) to remove any non-immune cytotoxicity associated with trastuzumab in the assay (EC Cytox with Tras).

ADCC in SKBR3 was calculated by subtracting (EC Cytox) from (EC Cytox with Tras).

% ADCC = (EC Cytox with Tras) – (EC Cytox)

**Direct cytotoxicity and trastuzumab-mediated ADCC in the presence of pembrolizumab.**

For the pembrolizumab assays, the same experimental set-up was used with the inclusion of additional, relevant basal cell death controls (EC:TC, 0:1 + pembrolizumab for K562 and SKBR3, and EC:TC, 0:1 + pembrolizumab + trastuzumab for SKBR3), and additional EC test conditions (EC: TC, 10:1 + pembrolizumab for K562 and SKBR3) and (EC: TC, 10:1 + pembrolizumab + trastuzumab for SKBR3).

Basal TC death adjusted direct PBMC cytotoxicity in the presence of pembrolizumab (EC Cytox P) was calculated by subtracting (EC:TC, 0:1 + pembrolizumab) from (EC: TC, 10:1 + pembrolizumab).

% Direct cytotoxicity (EC Cytox P) =

(EC: TC, 10:1 + pembrolizumab) – (EC:TC, 0:1 control + pembrolizumab)

Basal TC death adjusted EC cytotoxicity in the presence of trastuzumab and pembrolizumab was designated EC Cytox P with Tras and was calculated as follows:

% EC Cytox P with Tras =

(EC: TC, 10:1 + pembrolizumab + trastuzumab) –

(EC:TC, 0:1 + pembrolizumab + trastuzumab).

ADCC in the presence of pembrolizumab was calculated by subtracting (EC Cytox P) from (EC Cytox P with Tras):

% ADCC = (EC Cytox P with Tras) – (EC Cytox P)
