# Supplementary material for: Alterations in immune cell phenotype and cytotoxic capacity in HER2+ breast cancer patients receiving HER2-targeted neo-adjuvant therapy
Source: Br J Cancer. 2023 Jul 28;129(6):1022–31. doi: 10.1038/s41416-023-02375-y (PMC10491671; doi:10.1038/s41416-023-02375-y)

Minimum information about a flow cytometry experiment – Gaynor et al.

1. Experiment Overview

1.1. Purpose
The purpose of the experiment is to ascertain the association between peripheral blood mononuclear cells (PBMCs) and response to neo-adjuvant chemotherapy in HER2+ breast cancer by flow cytometric analysis of PBMCs stained with a standard set of immunophenotyping markers and PD-1.

1.2. Keywords

Blood, breast cancer, patient, leukocyte, lymphocyte, monocyte

1.3. Experiment Variables

Blood samples were taken from HER2+ breast cancer patients at baseline and post-6 cycles of chemotherapy (docetaxel/carboplatin) and HER2-targeted therapy (lapatinib or trastuzumab or the combination) using EDTA-containing blood tubes. The post treatment sample was taken 18-20 weeks after baseline sample. PBMCs were frozen in vials at 1 x 10^7 cells/mL in heat-inactivated FBS (Sigma Aldrich F9665) containing 5 % DMSO (Sigma Aldrich D2650). PBMCs were stored in liquid nitrogen. PBMCs were slowly revived and washed in pre-warmed RPMI 1640/10% HI FBS/0.5% pen/strep (Gibco 15140122). Revived PBMCs were incubated at 37 ºC for 4-5 hours before being checked for viability using Guava Viacount (Merck Millipore 4000-0041) on a Guava flow cytometer (Merck Millipore). N=39 pre-treatment samples and n=21 post treatment samples were available for phenotyping.

1.4. Organization

1.4.1. Name: Flow Cytometry Core Technology
1.4.2. Address: UCD Conway Institute, University College Dublin, Ireland

1.5. Primary Contact
1.5.1. Name: Dr Alfonso Blanco
1.5.2. Email Address: alfonso.blanco@ucd.ie

1.6. Date

PBMCs collected from 2011 – 2014
Flow cytometry analysis performed September 2018

1.7. Quality Control Measures

PBMCs were collected from all available patients including those who had a pathological complete response to treatment and those who did not. FMO controls were employed, and antibody titration optimisation was carried out where necessary. Quality Control of the Beckman Coulter CytoFLEX LX was performed using Daily QC and Daily IR Fluorospheres as per manufacturer specifications.

2. Flow Sample/Specimen Details

2.1. Sample/Specimen Material Description

Based on sample type, 2.1.1, 2.1.2, or 2.1.3 is required

2.1.1. Biological Samples

2.1.1.1. Biological Sample Description: PBMCs were isolated from 20-25ml peripheral blood. Blood was collected in EDTA blood tubes (BD Vacutainer #367525) and processed within 4 hours of blood draw. PBMCs were isolated using Ficoll-Paque (ThermoFisher 11778538)-based density centrifugation. PBMCs were frozen in vials at 1 x 10^7 cells/mL in heat-inactivated FBS (Sigma Aldrich F9665) containing 5 % DMSO (Sigma Aldrich D2650). PBMCs were stored in liquid nitrogen. PBMCs were thawed and counted as previously described in 1.3. PBMC samples were brought to a concentration of 3 x 10^6 cells/100 μL and processed for phenotyping in optimised IM Duraclone tubes (Beckman Coulter B53309) containing titrated PD-1 antibody (Biolegend B36123).

2.1.1.2. Biological Sample Source Description: Homo Sapiens

2.1.1.3. Biological Sample Source Organism Description:

2.1.1.3.1. Taxonomy: Homo Sapiens

2.1.1.3.4. Phenotype: < 18 years old, Gender: Female

2.1.1.3.5. Genotype: N/A

2.1.1.3.6. Treatment:

HER2+ breast cancer patients (n=88) were enrolled in the multi-centre, Phase II ICORG/CTRIAL-IE 10-05/TCHL/NCT01485926 neoadjuvant clinical trial. Patients were randomised to one of three trial arms to receive docetaxel (T) (75 mg/m2), carboplatin (C) (AUC 6) and trastuzumab (H) (8 mg/kg loading dose and 6 mg/kg for subsequent six cycles) and/or lapatinib (L) (1000 mg daily until 1 week prior to surgery). GCSF was mandatory for all patients as primary prophylaxis for febrile neutropenia while receiving chemotherapy, as was a prophylactic steroid regimen (dexamethasone) prior to each dose of docetaxel. Following surgery, patients received 1 year of trastuzumab from first dose of trastuzumab. The TCL arm was discontinued early due to preliminary results of the ALTTO trial which showed lapatinib alone to be inferior to trastuzumab, or trastuzumab and lapatinib in relation to patient survival outcomes. The primary endpoint was to determine efficacy of TCH, TCL and TCHL in the neoadjuvant treatment of HER2+ breast cancer using pCR. Secondary endpoints were to assess the clinical response rate and overall response rate by treatment arm and to investigate potential markers of response to trastuzumab- and lapatinib-based chemotherapy. Blood samples were taken from patients before cycle 1 of treatment (pre-treatment) and after they completed cycle 6 of neo-adjuvant treatment (post-treatment), but before surgery. pCR was defined as no residual invasive tumour in the breast or lymph nodes at surgery. The ICORG/CTRIAL-IE 10-05 study protocol consented patients for blood samples to be taken. Healthy volunteer blood sample ethical approval was obtained from Dublin City University Ethics Committee.

2.2. Sample Treatment(s) Description

PBMCs were isolated, thawed and counted as previously described.
PBMC samples were brought to a concentration of 3 x 10^6 cells/100 μL.

An optimised DURAClone IM Phenotyping Basic kit (Beckman Coulter B53309) was used to phenotype cell types. Titration and compensation was carried out for PD-1 antibody (Biolegend B36123) using PBMCs from patients and healthy volunteers. Single stained controls were used for compensation. FMO controls were included for gating purposes.
PBMCs were incubated with 2.5 µg/1 x 10^6 cells of human Fc block (BD Biosciences 564219). 100 µL
PBMCs were added to a sample tube of DURAClone IM Phenotyping Basic kit followed by PD-1 antibody, along with a zombie viability dye (BioLegend B423107).
Red blood cell lysis reagent (Beckman Coulter A09777) was added to the tube.
The tubes were then incubated in the dark for 15 minutes at room temperature.
The cells were centrifuged and resuspended in PBS containing 0.8% fixative solution (Beckman Coulter 8546859).
Samples were stored overnight at 4 °C and analysed using the CytoFLEX LX (Beckman Coulter).
Analysis was carried out using FCS Express 6.06.0022 software. Gating utilised FMO controls and the recommended DURAClone IM Phenotyping Basic kit gating strategy (Beckman Coulter).

2.4. Fluorescence Reagent(s) Description

Each sample has been stained with reagents according to the following table:

Laser	EM Filter	Marker	Color / Format	Characteristic	Host / Target	Isotype	Clone	Company	Catalog
488	525/40	CD16	FITC	NK cell lineage	Mouse anti-Human	IgG1	IgG1	DURAClone IM PhenoBasic B53309	
561	585/42	CD56	PE	NK cell lineage	Mouse anti-Human	IgG1	IgG1		
561	610/20	CD19	ECD	B cell lineage	Mouse anti-Human	IgG1	IgG1		
561	763/43	CD14	PE-Cy7	Monocyte lineage	Mouse anti-Human	IgG2a	IgG2a		
638	660/10	CD4	APC	T Helper cell lineage	Mouse anti-Human	IgG1	IgG1		
638	712/25	CD8	Alexa Fluor 700	Cytotoxic T cell lineage	Mouse anti-Human	IgG1	IgG1		
638	763/43	CD3	APC-Alexa 750	T cell lineage	Mouse anti-Human	IgG1	IgG1		

Laser	EM Filter	Marker	Color / Format	Characteristic	Host / Target	Isotype	Clone	Company	Catalog
405	525/40	CD45	Krome Orange	Leukocyte gating	Mouse anti-Human	IgG1	IgG1		
488	690/50	CD279 (PD1)	PC5.5	Immune suppression marker	Mouse anti-Human	IgG1	IgG1	Biolegend	B36123
360	450/45	Zombie UV dye	UV dye	Live/Dead cell marker				Biolegend	423107

3. Instrument Details
3.1. Instrument Manufacturer
Beckman Coulter
https://www.beckmancoulter.com/

3.2. Instrument Model
CytoFLEX LX

3.3. Instrument Configuration and Settings

3.3.1. Optical Filters
The instrument has not been altered; fixed-alignment cuvette flow cell.
Instrument date of manufacture: 2017

4. Data Analysis Details
4.1. List-mode Data File
FCS data files can be obtained by contacting Dr Alfonso Blanco after this work has been published.
4.2. Compensation Details
Compensation has been performed computationally post acquisition according to the following spillover matrix (values in %):

Autofluorescence	Channel	B525-FITC%	Y585-PE%	Y610 mCHERRY%	Y710-PC5.5%	Y763-PC7%	R660-APC%	R712-APCA700%	R763-APCA750%	V525-KrO%	NUV525%
2.78	**B525-FITC**		0.82	0.22	0.29	0.98	0	0.02	0	1.57	0
0.97	**Y585-PE**	0		11.79	2.92	6.7	0.02	0.02	0.04	0	0
2.01	**10 mCHER**	0	85.22		2.64	6.04	0.88	0.04	0.47	0	0
0.39	**Y710-PC5.**	0	0	0		0.06	0.01	0.1	0.2	0	0
0.09	**Y763-PC7**	0	6.39	4.62	3		2.64	8.53	0.85	0	0
0.43	**R660-APC**	0	0.01	0.06	1.84	0.11		0.37	28	0.03	0
0.18	**'12-APCA7**	0	0.74	0.04	0	0.4	35.71		11.21	0.07	0
1.82	**'63-APCA7**	0	0	0	7.02	1.05	7.64	24.02		0.07	0
5.97	**V525-KrO**	1.17	1.01	0	0	0	0	0	0		0
0	**NUV525**	0	0	0	0	0	0	0	0	0	

4.3. Data Transformation Details
4.3.1. Purpose of Data Transformation
Visualization and gating.
4.3.2. Data Transformation Description

FCS Express 6.06.0022 software (De Novo Software) default visualization settings have been used for gating. All fluorescence parameters were displayed with logarithmic scaling:

4.4. Gating (Data Filtering) Details

4.4.1. Gate Description

Analysis was carried out using FCS Express 6.06.0022 software. Gating utilised FMO controls and the recommended DURAClone IM Phenotyping Basic kitgating strategy (Beckman Coulter).
The gating strategy involves the following gates:

- FSC-SSC gate to define singlet population
- CD45 gate applied to define the leukocytes.
- FSC-Zombie dye gate applied on leukocytes to discriminate viable leukocytes.
- CD3+, CD19+ and CD56+ populations were identified within the singlet CD45+ lymphocyte population.
- CD4+ and CD8+ cells were identified within the CD3+ population.
- CD56+ and CD19+ populations excluded CD3+ cells.
- CD14+ cells were separated from the CD45+ leukocyte populations based on side scatter and CD14 expression.
- CD16 expression was examined within the CD56+ and CD14+ parent populations, using quadrants to differentiate CD16+ populations (Upper right) and CD16- populations (lower right).
- PD-1 expression was collectively examined across the combined CD45+ lymphocyte and monocyte populations, or within the specific cell subpopulations highlighted in the study.

4.4.2. Gate Statistics

The following table shows percentages of each of the subpopulations defined by described gates for an example sample:

Filename	Gate	# of events	% of all cells
3B.fcs compensated	None	1000000	100
3B.fcs compensated	Singlets	970226	97.02
3B.fcs compensated	CD45+	568221	56.82
3B.fcs compensated	Lymphocyte	502269	50.23
3B.fcs compensated	Leukocyte	568221	56.82
3B.fcs compensated	CD14+	33393	3.34
3B.fcs compensated	CD3+	228368	22.84
3B.fcs compensated	CD8+	79975	8.00
3B.fcs compensated	CD4+	118880	11.89
3B.fcs compensated	CD19+	17038	1.70
3B.fcs compensated	CD56+	43756	4.38
3B.fcs compensated	CD279+	15455	1.55
3B.fcs compensated	CD14+/CD279+	51	0.01
3B.fcs compensated	CD56+/CD279+	6903	0.69
3B.fcs compensated	CD8+/CD279+	1760	0.18
3B.fcs compensated	CD56+/CD16+	10943	1.09
3B.fcs compensated	CD56+/CD16-	21075	2.11
3B.fcs compensated	CD56+/CD16+/CD279+	1199	0.12
3B.fcs compensated	CD56+/CD16-/CD279+	6497	0.65
3B.fcs compensated	CD14+/CD16+	1489	0.15
3B.fcs compensated	CD14+/CD16-	31904	3.19
3B.fcs compensated	CD14+/CD16+/CD279+	41	0.00
3B.fcs compensated	CD14+/CD16-/CD279+	388	0.04

4.4.3. Gate Boundaries *(or images or detailed gate membership information)*

Examples of gating boundaries for Sample 3B. CD279 = PD-1.

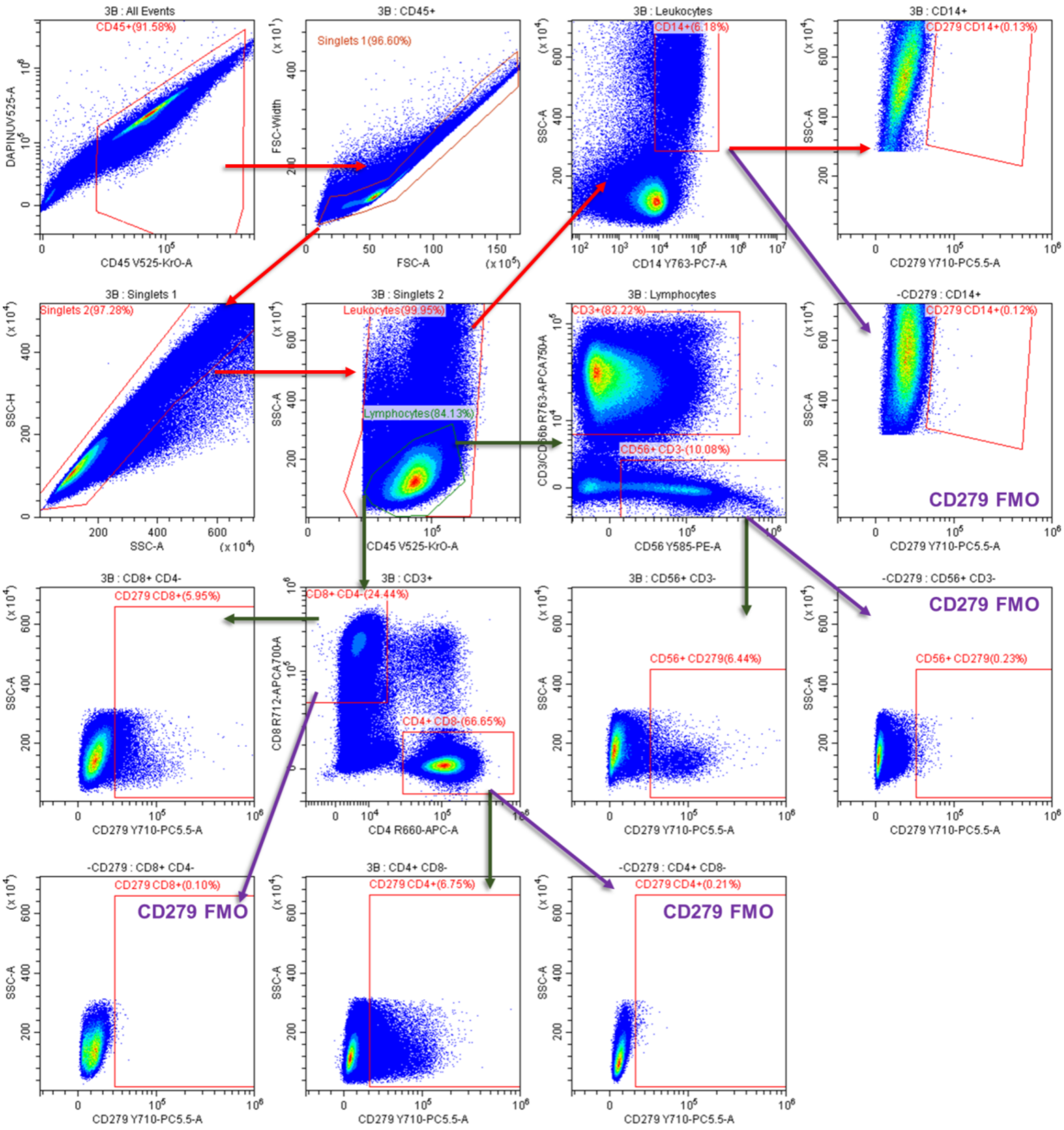

Supplement: Supplementary file 3 — Minimum information about a flow cytometry experiment [file 41416_2023_2375_MOESM3_ESM.pdf]
